# Pre-cleaned bare wooden toothpicks for the determination of drugs in oral fluid by mass spectrometry

**DOI:** 10.1007/s00216-022-03977-w

**Published:** 2022-03-10

**Authors:** Jaime Millán-Santiago, Rafael Lucena, Soledad Cárdenas

**Affiliations:** Affordable and Sustainable Sample Preparation (AS2P) Research Group, Departamento de Química Analítica, Instituto Universitario de Investigación en Química Fina Y Nanoquímica (IUNAN), Universidad de Córdoba, Campus de Rabanales, Edificio Marie Curie, 14071 Córdoba, Spain

**Keywords:** Sample treatment, Wooden toothpicks, Therapeutic drugs, Oral fluid, Mass spectrometry

## Abstract

This article deepens the potential of pre-cleaned bare wooden toothpicks (pb-WTs) for extracting drugs (antidepressants and acetaminophen) from oral fluid samples. The leaching of the intrinsic compounds from the wood matrix is identified as the main challenge for the final determination of the targets, even when a very selective instrumental technique, such as mass spectrometry, is employed. The pre-cleaning of the WTs is proposed for improving the analytical performance. The number of cleaning cycles depends on the injection mode (direct infusion or chromatography) into the mass spectrometer. The different variables affecting the extraction of selected antidepressant drugs were studied in detail, and the optimum procedure was validated using the two mentioned injection modes. The limits of detection were in the ranges 0.1–0.5 ng/mL and 0.1–0.3 ng/mL for direct infusion and liquid chromatography, respectively. The intra-day precision (expressed as relative standard deviation) was better than 12.1% and 8.6%, for direct infusion and liquid chromatography, respectively. Single-blind samples were used to study the applicability of the method. Finally, as a proof-of-concept, the potential of pb-WTs for in vivo sampling was outlined.

**Graphical abstract:**

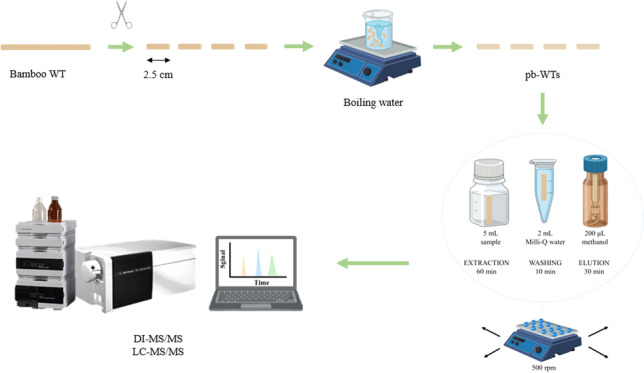

**Supplementary Information:**

The online version contains supplementary material available at 10.1007/s00216-022-03977-w.

## Introduction

White Analytical Chemistry (WAC) is a recently introduced concept which updates and complements the Green Analytical Chemistry (GAC) principles [1]. WAC establishes that new methods must balance sustainability with analytical usefulness. Although sample preparation is identified by GAC as a procedure with high potential environmental impact, it is accepted in the GAC-WAC context as it is essential to solving many analytical problems. Microextraction plays a pivotal role in sample preparation, and it involves the simplification and miniaturization of the techniques, thus reducing their environmental impact. As the tenth principle of GAC suggests, this impact can be further reduced by using reagents and materials obtained from renewable sources [2].

Natural materials are promising tools in microextraction due to their biodegradability, sustainability, and eco-friendliness character [3–5]. They are commonly modified to promote interaction with the target compounds. These modifications involve their superficial coating with polymers (e.g., polymeric ionic liquids (PILs) [6], molecularly imprinted polymers (MIPs) [7], commercial ones [8], nanoparticles [9], nanocomposites [10], and silanes [11]. Although the modification implies several advantages, bare biosorbents simplify and speed up the workflow and reduce the environmental impact related to their preparation. For this purpose, different unmodified biosorbents have been used in microextraction [12–15]. However, they require a previous cleaning to remove impurities or intrinsic components that may interfere in the instrumental determination of the target compounds [16, 17].

Wood emerges as an alternative sorbent for the extraction of analytes from complex samples. It is composed of cellulose, hemicellulose, lignin, and other minority intrinsic compounds. Due to its chemical composition, wood can establish hydrogen bonds and π-π interactions with target compounds of different polarities. Wooden toothpicks (WTs) are the most common extraction format, although wooden sticks [18] and wooden capillaries [19] have also been reported. Wooden-based sorbents also present rigidity and porosity. The versatility of WTs allows different sampling modalities including (i) sample deposition [20–26], (ii) dipping of liquid samples [27, 28], (iii) puncturing solid samples [29, 30], (iv) adhesion of granule/powdered samples [28, 31–35], and (v) conventional extraction-elution-analysis workflow [18, 36–41]. Moreover, the biocompatibility of wood derived from its lignocellulosic nature opens the door to in vivo sampling. In vivo sampling-extraction simplifies the analytical process minimizing the cross-contamination between samples and avoiding losses of chemical information during the sample storage/transportation [42–44]. An ideal in vivo technique should be affordable, cheap, and portable, with low solvent requirements, and should allow the isolation/preconcentration of the analytes [45].

The narrow shape of WTs permits their direct coupling to ambient ionization mass spectrometry (AIMS) in the so-called wooden-tip electrospray mass spectrometry (WT-ESI–MS) technique [46]. Although WT-ESI–MS is, by far, the most common technique when using WTs, direct infusion tandem mass spectrometry (DI-MS/MS) [41] and liquid chromatography-tandem mass spectrometry (LC–MS/MS) [18] have also been used.

In this article, pre-cleaned bare WTs (pb-WTs) have been successfully used to determine six tricyclic antidepressants (TCAs) in oral fluid samples by LC–MS/MS and DI-MS/MS. The effect of the pre-cleaning cycles, which only require boiled tap water, on the performance of both analytical techniques has been studied in-depth. The reduction of intrinsic interferents originating from the wood matrix decreases the ion suppression, enhancing the analytical sensitivity. The extraction workflow allows the simultaneous processing of multiple samples providing a sample throughput up to 6.5 and 12 samples/h for LC–MS/MS and DI-MS/MS, respectively. This aspect demonstrates the potential implementation of the method in routine laboratories. The proposed approach permits the analysis of human oral fluid samples as the linear range fits into the expected concentration of TCAs in this matrix. Single-blind spiked samples were used to demonstrate the applicability of the method. The potential of pb-WTs for in vivo sampling has been finally outlined using acetaminophen as a proof-of-concept analyte.

## Materials and methods

### Reagents

All the reagents were of analytical grade or better. Unless otherwise indicated, they were purchased from Sigma-Aldrich (Madrid, Spain). Stock standard solutions of the analytes (amitriptyline (AMI), clomipramine (CLO), desipramine (DES), imipramine (IMI), nortriptyline (NOR), and trimipramine (TRI)) were prepared at a concentration of 2000 mg/L in methanol (PanReac, Barcelona, Spain) and stored at – 20 °C. Acetaminophen was used as model analyte for in vivo sampling.

Clomipramine-*d*_3_ (CLO-*d*_*3*_), desipramine-*d*_3_ (DES-*d*_*3*_), and nortriptyline-*d*_*3*_ (NOR-*d*_*3*_) were used as internal standards (IS) in LC–MS/MS. IS stock standard solutions were prepared at a concentration of 100 mg/L in methanol and stored at – 20 °C. The signal of each analyte was corrected by its corresponding deuterated IS. The signal of TRI, IMI, and AMI was corrected using DES-*d*_*3*_ as no deuterated compounds were at our disposal.

Working solutions were prepared by diluting the stock solutions in methanol or Milli-Q water (Millipore Corp., Madrid, Spain) as required.

Methanol, Milli-Q water, and formic acid were used as components of the mobile phase in LC–MS/MS and DI-MS/MS analysis.

Bamboo WTs were purchased in a local market in Córdoba (Spain).

### Oral fluid collection

The oral fluid blank samples used during the optimization were obtained using a Salivette® sample collection device (Sarstedt, Nümbrecht, Germany) following the procedure described in a previous publication of the group [41], with slight modifications. For clarity, the procedure is described in detail in the supplementary information. However, for the analysis of real samples, direct oral fluid spitting is recommended because of the retention ability of the cellulosic cotton roll towards the target analytes [47, 48]. Salivette® was used only during the optimization since it provides a large volume of samples more easily. Also, blank samples (obtained from a healthy volunteer who was not medicated with TCAs) were needed in the optimization/validation, so Salivette was fully compatible.

### Preparation of the sorbents

Bamboo WTs (o.d. 2.3 mm) were cut in segments of 2.5 cm in length. The cut WTs were introduced into a 600-mL beaker with 400 mL of boiling water under magnetic agitation for 1 h. This process was repeated with fresh boiling water as demanded. Finally, the pb-WTs were dried at room temperature until their final use. Although other washing approaches (e.g., using overheated water or ultrasounds) are potentially applicable, the proposed approach is simple, affordable, and easy to develop in any laboratory.

### Extraction procedure

**Fig. 1:**
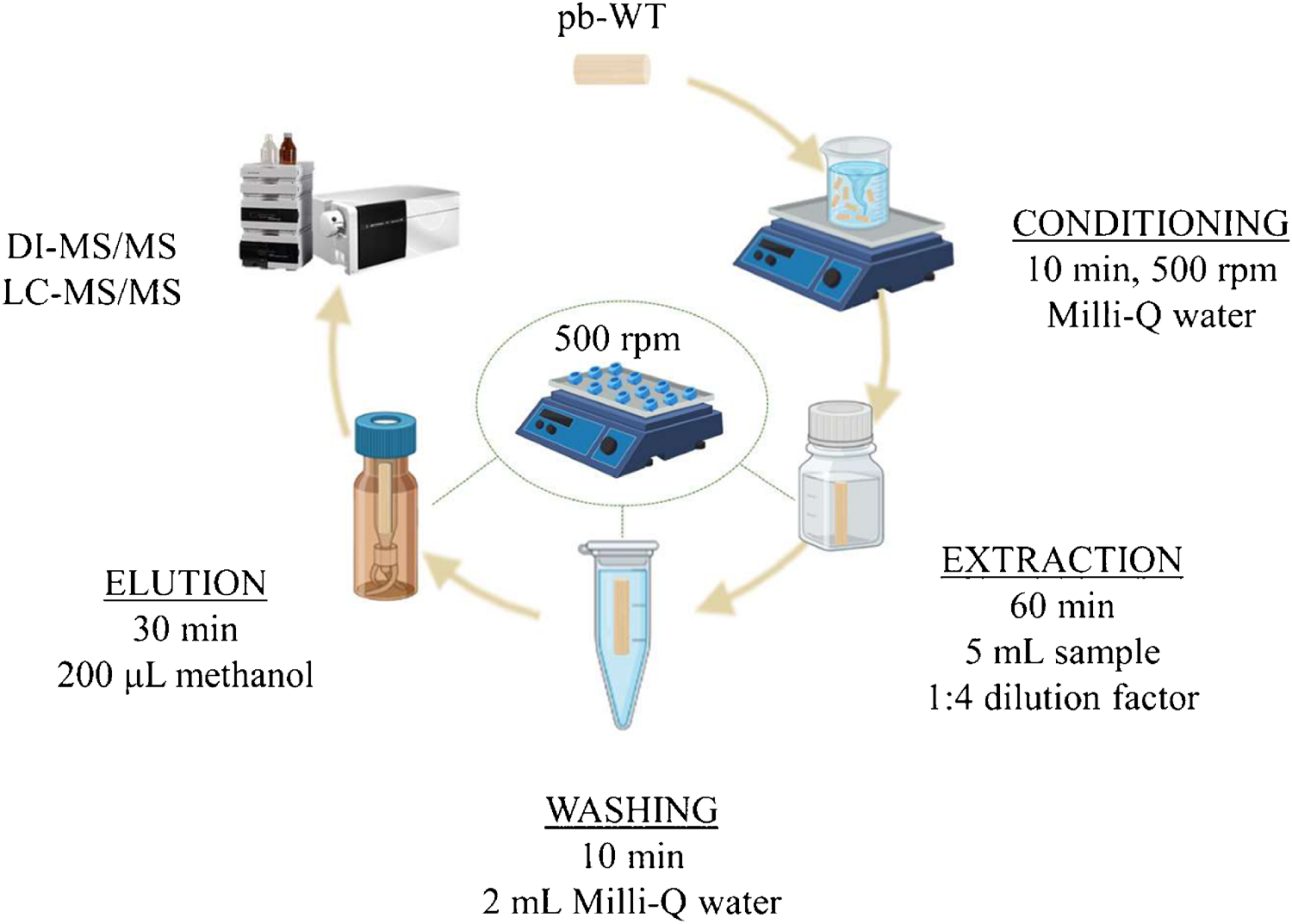
Extraction procedure showing the main steps: conditioning, extraction, washing, and elution

The extraction procedure is depicted in Fig. 1. The pb-WTs were conditioned in Milli-Q water under magnetic agitation. Subsequently, pb-WTs were individually transferred into a glass vial containing 5 mL of the 1:4 diluted sample. The length of the sorbents allows for their entire immersion in the sample to maximize the practical surface area of the materials. The samples (up to 25, although the system allows the simultaneous extraction of 50) were simultaneously stirred in an orbital stirrer (Vibramax 110, Heidolph, Schwabach, Germany) for 1 h at 500 rpm. After the extraction step, each pb-WTs was washed with 2 mL of Milli-Q water in an Eppendorf tube (10 min, 500 rpm) to remove potential matrix interferences. The pb-WTs enriched with the analytes were dried with a tissue and transferred into an HPLC vial. The analytes were finally eluted with 200 μL of methanol for 30 min at 500 rpm. 5 µL of the eluate was analyzed by LC–MS/MS and DI-MS/MS for the identification and quantitation of the analytes.

The optimum pre-cleaning cycles depend on the final analysis strategy. Two cycles are used in LC–MS/MS, while 5 cycles are needed in DI-MS/MS.

### LC–MS/MS and DI-MS/MS analysis

LC–MS/MS analyses were performed on an Agilent 1260 Infinity HPLC system (Agilent, Palo Alto, CA, USA) equipped with a binary high-pressure pump for mobile phase delivery and an autosampler. A guard column (0.2-μm filter, 2.1 mm) was employed to prevent the MS from potential particles. Chromatographic separation was carried out by reversed-phase using an Eclipse Plus C18 (4.6 × 100 mm, 3.5 μm) column. The mobile phase consisted of (A) aqueous solution with 0.1% (v/v) formic acid and (B) methanol, following an isocratic separation with a solvent ratio of 10:90 (v/v) for 7 min. DI-MS/MS analyses were performed in the absence of column using the same solvent ratio with a sample analysis of 1.5 min. The injection volume was 5 μL and the flow rate was maintained constant at 0.2 mL/min in both modalities. MS parameters are shown in Table S1.

## Results and discussion

### Effect of the WT pre-cleaning on the analytical signal

The use of bare lignocellulosic materials as sorbents is challenging since they present compounds that may leach during extraction or elution. These compounds can interfere not only with the extraction of the analytes, but also with their instrumental determination. In this sense, they are usually washed before use.

In the case of WTs, this situation is even more complicated as these materials have been mainly used in combination with direct MS analysis. The leaching of these intrinsic components hinders the efficient ionization of the analytes affecting the sensitivity of the method. This effect was evaluated in-depth in this article. For doing so, WTs were incubated with pure water following the procedure described in the supplementary information. After the incubation, the supernatant was spiked with the target analytes at 20 μg/L, and it was analyzed by DI-MS/MS. The results, including the signal provided by a standard prepared in Milli-Q at the same concentration for comparative purposes, are shown in Fig. S1. As can be observed, the presence of leached compounds from the wood matrix negatively affects to the ionization of the target compounds, thus resulting in a lower analytical signal. This point was evaluated in-depth considering the whole extraction procedure (isolation/elution) that it is described below.

**Fig. 2:**
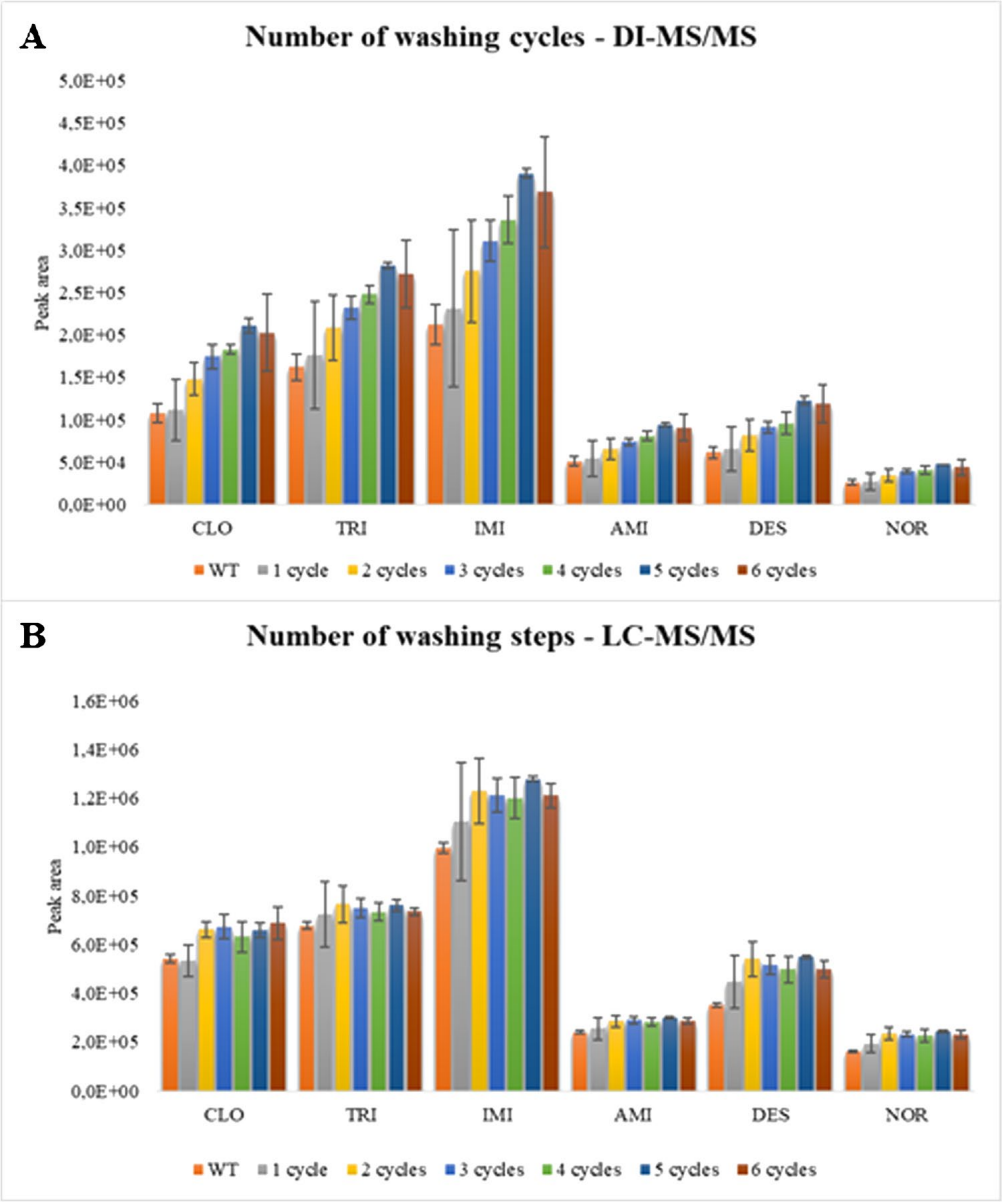
Effect of the washing cycles in the extraction of the analytes by **A** DI-MS/MS and **B** LC–MS/MS

The pre-cleaning of the WTs in water was considered an environmentally friendly strategy to avoid this shortcoming. For this purpose, the WTs were washed several times in boiling water, as reported in “Preparation of the sorbents,” and finally used to extract the analytes from aqueous standards. The eluates obtained in the extraction were analyzed by DI-MS/MS. As shown in Fig. 2A, the analytical signal for the analytes increased with the number of cleaning cycles. This aspect also benefits the spectrometer that is less exposed to dirty. Attending to the results, 5 pre-cleaning cycles were selected for DI-MS/MS.

The use of a chromatographic separation can also reduce ion suppression in MS. For this reason, the effect of the pre-cleaning cycles using LC–MS/MS as the instrumental technique was also evaluated. As is shown in Fig. 2B, the effect of the cycles on the signals is less pronounced when LC–MS/MS is used. In this case, 2 pre-cleaning cycles were selected as the optimum value. However, the instrument is more exposed in such conditions as the total number of co-eluted compounds is higher, although they do not influence the signal.

This reduction of the leaching effect opens the door to two different modalities: using pb-WTs with 2 washing cycles in LC–MS/MS or pb-WTs with 5 washing cycles in DI-MS/MS. The first approach implies longer analysis times (7 min/sample) but a more rapid preparation of the sorbent. The second approach implies faster sample analysis (1.5 min) but entails a longer sorbent preparation.

### Effect of the different variables that affect the extraction

The variables that are involved in the extraction efficiency of the analytes were evaluated following a univariate approach using aqueous standards containing the TCAs and ISs at 20 ng/mL. In this case, the investigated variables were sample pH, ionic strength, and extraction time.

**Fig. 3:**
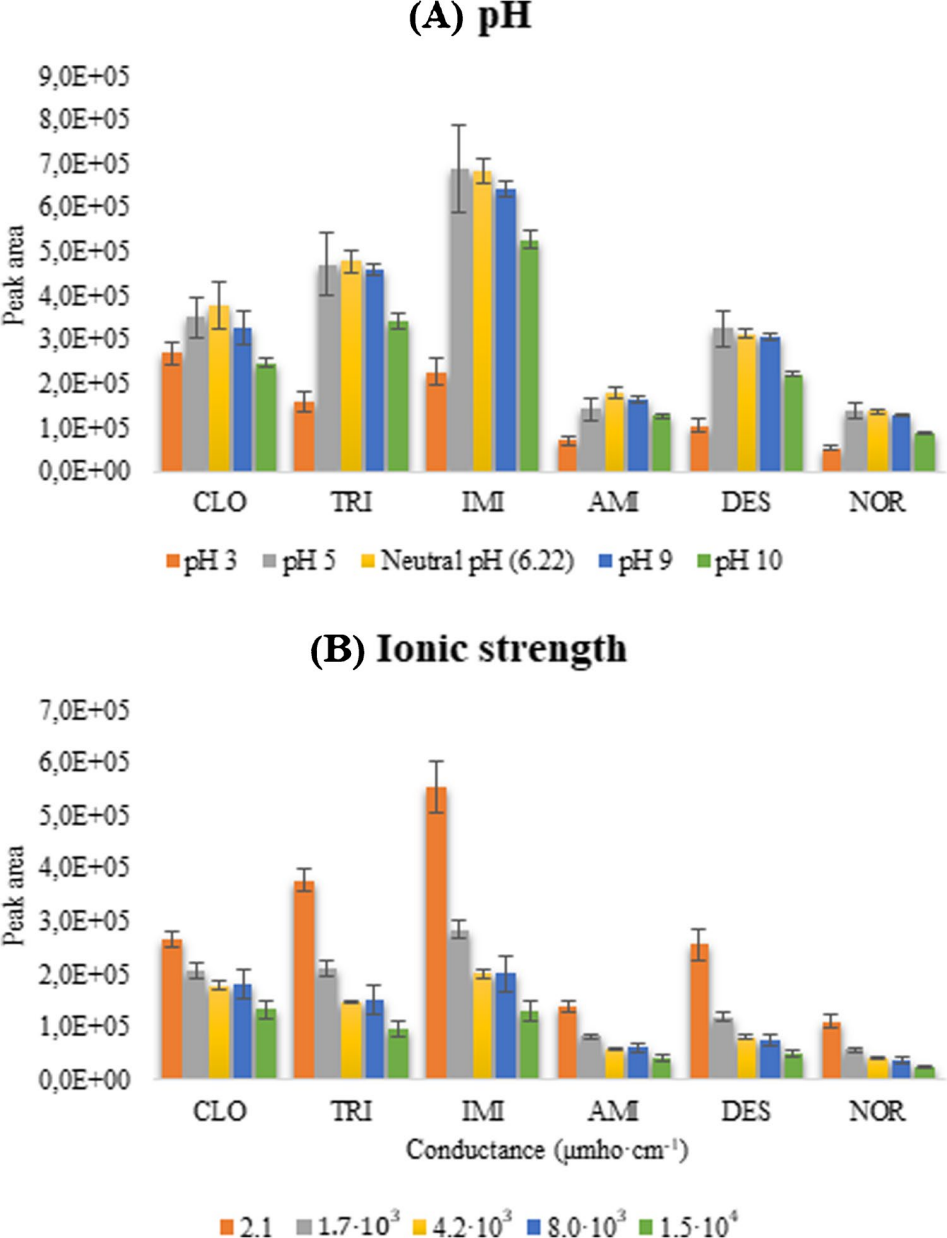
**A** Effect of the pH in the extraction of the analytes. **B** Effect of the ionic strength in the extraction of the analytes. The peak area is obtained by the LC–MS/MS injection mode

Firstly, the effect of the sample pH was studied in the interval from 3 to 10. More alkaline values were not evaluated to avoid the eventual damage of the WTs. The obtained results, expressed as absolute signal (peak area for each analyte), are shown in Fig. 3A. The extraction was favored at the physiological pH of the saliva (6–7) and, consequently, the pH adjustment of the samples is not mandatory.

The ionic strength can affect the extraction process in terms of kinetics (increasing viscosity diminish the mass transference) and thermodynamics (salting-out effect). The effect of the ionic strength was evaluated with the addition of NaCl to aqueous standards ranging from 0 to 1% (w/v). The conductance of the solutions was measured in a conductometer, and the resulting values ranged from 2.1 to 1.5·10^4^ μmho/cm. Figure 3B represents the negative effect of the increasing salinity in the extraction of the analytes expressed as the absolute signal. However, the relative signal (corrected by the IS) is less affected by the ionic strength, making the adjustment not necessary.

As the matrix of the sample can also affect the extraction kinetics, the extraction time was studied using a pool of different blank saliva samples spiked with the analytes. The samples were diluted to minimize the negative effect of the viscosity in the extraction kinetics. A 1:4 dilution factor was selected according to our previous studies [41]. The studied interval spans from 5 to 60 min. Further extraction times were not evaluated to provide a high sample throughput. The extraction time had a positive effect on the extraction of the analytes both in aqueous standards (Fig. S2A) and spiked blank samples (Fig. S2B), and the equilibrium is not reached for 60 min. The extraction time was established at 60 min to guarantee the sensitivity of the method. This time does not affect the sample throughput too much because multiple samples can be extracted simultaneously.

### Method validation

The analytical method was validated in terms of linearity, limit of detection (LOD), limit of quantification (LOQ), accuracy, and precision (inter and intra-day in both cases) using matrix-matched calibration curves for each analyte. The analytical method was validated, under optimized conditions, using diluted saliva with ultrapure Milli-Q water in a 1:4 dilution factor and fortified with known concentrations of each analyte. The validation was carried out by two different modalities: DI-MS/MS (Table 1) and LC–MS/MS (Table 2) using pb-WTs purified with 5 and 2 washing cycles, respectively. The linearity, with *R*^2^ > 0.9976, demonstrates an appropriate correlation. The linear range was calculated at seven different concentration levels ranging from 0.4 to 800 ng/mL, considering the dilution of the spiked samples. The LODs were calculated as 3 times the standard deviation of the intercept divided by the slope of the calibration graph. The LOQs were calculated as 10 times the standard deviation of the intercept divided by the slope of the calibration graph. Precision, determined as the relative standard deviation (RSD), was calculated to estimate the variation between different replicates. Intra-day precision and inter-day precision were calculated spiking the analytes at three different concentrations (4, 80, and 400 ng/mL), analyzing triplicates at three different days. Intra-day and inter-day precision, calculated at three different concentration levels, were lower than 8.6% and 14.3%, respectively, for LC–MS/MS, and lower than 9.3% and 16.4% for DI-MS/MS, respectively. Accuracy, expressed as relative recovery, was calculated on three different days, analyzing three levels of concentration (4, 80, and 400 ng/mL) by triplicate, varying between 77–121% and 94–133% for LC–MS/MS and DI-MS/MS, respectively.

**Table 1:** Analytical figures of merit for the DI-MS/MS method

Analyte	Linear range (ng/mL)	LOD (ng/mL)	LOQ (ng/mL)	*R*^2^	Intra-day precision (*n* = 3, %)			Inter-day precision (*n* = 9, %)			Relative recovery (*n* = 9, %)		
					4 (ng/mL)	80 (ng/mL)	400 (ng/mL)	4 (ng/mL)	80 (ng/mL)	400 (ng/mL)	4 (ng/mL)	80 (ng/mL)	400 (ng/mL)
Clomipramine	0.4–800	0.1	0.4	0.9996	4.2	0.7	0.7	16.4	9.3	6.9	96–133	100–120	97–111
Trimipramine	1.6–800	0.5	1.6	0.9998	9.3	4.4	5.3	12.0	11.1	11.4	94–120	98–122	98–123
Imipramine	1–800	0.3	1.0	1	6.8	2.4	12.1	15.6	12.4	10.8	95–133	100–127	99–122
Amitriptyline	0.7–800	0.2	0.7	0.9995	6.6	2.3	3.6	15.2	10.1	9.6	95–132	100–121	98–118
Desipramine	0.3–800	0.1	0.3	0.9999	3.1	0.6	2.0	14.6	5.7	5.9	98–133	103–115	99–110
Nortriptyline	0.7–800	0.2	0.7	0.9996	6.7	3.7	1.4	11.7	9.8	6.4	98–127	100–123	101–113

*LOD*, limit of detection; *LOQ*, limit of quantification. Precision values are expressed as relative standard deviation

**Table 2:** Analytical figures of merit for the LC–MS/MS method

Analyte	Linear range (ng/mL)	LOD (ng/mL)	LOQ (ng/mL)	*R*^2^	Intra-day precision (*n* = 3, %)			Inter-day precision (*n* = 9, %)			Relative recovery (*n* = 3, %)		
					4 (ng/mL)	80 (ng/mL)	400 (ng/mL)	4 (ng/mL)	80 (ng/mL)	400 (ng/mL)	4 (ng/mL)	80 (ng/mL)	400 (ng/mL)
Clomipramine	0.3–800	0.1	0.3	0.9997	2.3	0.7	1.6	14.3	5.8	7.5	84–113	97–110	98–112
Trimipramine	1.1–800	0.3	1.1	0.9976	8.6	3.2	6.3	13.5	6.8	7.8	81–107	90–102	89–103
Imipramine	0.5–800	0.2	0.5	0.9994	8.1	1.5	4.1	11.9	6.9	7.7	86–111	94–108	94–108
Amitriptyline	0.5–800	0.2	0.5	0.9986	5.4	1.8	4.7	16.2	6.6	7.5	77–106	92–103	91–105
Desipramine	0.6–800	0.2	0.6	0.9997	2.5	1.8	1.6	14.1	7.8	8.3	82–111	99–116	96–112
Nortriptyline	0.3–800	0.1	0.3	0.9994	3.2	1.4	0.5	8.0	8.2	7.3	84–99	102–121	101–114

*LOD*, limit of detection; *LOQ*, limit of quantification. Precision values are expressed as relative standard deviation

### Analysis of single-blind samples

The analysis of single-blind samples was carried out to evaluate the feasibility of the proposed method in analyzing samples with an unknown concentration of the analytes. These blind samples were prepared by the second author of the article and analyzed by the first author. In this case, three different spiked samples were analyzed by LC–MS/MS and DI-MS/MS, showing promising results in terms of qualitative and quantitative analyses (see Table 3). Both methodologies provided 100% of success in the identification of the compounds. Also, the quantitative analysis reported relative recoveries spanning from 69 to 89% for LC–MS/MS and from 69 to 85% for DI-MS/MS.

**Table 3:** Analysis of blind samples by DI-MS/MS and LC–MS/MS methods

Sample number	Spiked analyte	Spiked concentration (ng/mL)	DI-MS/MS		LC–MS/MS	
			RSD (*n* = 3, %)	Recovery (*n* = 3, %)	RSD (*n* = 3, %)	Recovery (*n* = 3, %)
**1**	Trimipramine	100	2.7	73	1.1	80
	Imipramine	400	1.6	79	0.7	78
	Amitriptyline	160	0.6	69	1.0	68
	Desipramine	40	1.1	69	0.3	66
**2**	Clomipramine	400	3.3	85	0.6	88
	Imipramine	100	0.4	74	0.7	79
	Desipramine	200	1.7	70	0.8	74
	Nortriptyline	60	4.0	72	2.3	69
**3**	Clomipramine	120	1.1	79	0.3	77
	Trimipramine	160	2.9	74	4.7	76
	Amitriptyline	20	1.1	78	5.3	89
	Nortriptyline	200	3.2	81	0.5	80

*RSD*, relative standard deviation

Jaime Millán-Santiago^1^, Rafael Lucena^1^, Soledad Cárdenas^1,*^

## Conclusions and perspectives

Natural materials present great potential as sorbents in microextraction. Although they can be modified to boost the extraction capacity and/or selectivity, bare materials are environmentally friendly sorbents. In this article, pb-WTs have been evaluated as sorbents for isolating drugs from the oral fluid. The pre-cleaning of the WTs in water is essential to improve the analytical performance in MS under two injection modes (direct infusion and liquid chromatography). The direct infusion was more sensitive to co-eluted compounds from the wood matrix, requiring a larger number of pre-cleaning cycles. However, it provides a more rapid analysis compared to LC, also reducing the organic solvents required for each analysis.

The low price of WTs permits the cheap preparation of multiple extraction devices that can be disposed after being used. This aspect is also relevant from the bioanalytical point of view. It minimizes the exposure of the operators to contaminated devices and avoid cross-contamination as each sample is extracted with a new device.

WTs, especially if they are washed using pure water, are biocompatible thanks to their lignocellulosic nature. The potential use of this biocompatible material for in vivo sampling arose as a natural hypothesis during this project. This hypothesis has been outlined using acetaminophen as model analyte because positive samples for TCAs (ideal for this evaluation) were not finally available. Acetaminophen does not require a medical prescription, and it is used as a common pain reliever allowing the easy collection of positive samples . In this preliminary study, explained in detail in the supplementary information, pb-WTs provided promising results on extracting acetaminophen from the oral cavity allowing to follow the absorption profile of the drug (Fig. S3). This line will be further studied in future works.

## Supplementary Information

Below is the link to the electronic supplementary material.

Supplementary file1 (DOCX 175 KB)
