# Supplementary material for: Pre-cleaned bare wooden toothpicks for the determination of drugs in oral fluid by mass spectrometry
Source: Anal Bioanal Chem. 2022 Mar 10;414(18):5287–96. doi: 10.1007/s00216-022-03977-w (PMC9242915; doi:10.1007/s00216-022-03977-w)
Supplement: Supplementary file 1 — Supplementary file1 (DOCX 175 KB) [file 216_2022_3977_MOESM1_ESM.docx]

**SUPPLEMENTARY INFORMATION**

**Pre-cleaned bare wooden toothpicks for the determination of drugs in oral fluid by mass spectrometry**

Jaime Millán-Santiago, Rafael Lucena, Soledad Cárdenas*

Affordable and Sustainable Sample Preparation (AS_2_P) Research Group, Departamento de Química Analítica, Instituto Universitario de Investigación en Química Fina y Nanoquímica (IUNAN), Universidad de Córdoba, Campus de Rabanales, Edificio Marie Curie, E-14071 Córdoba, España.

Corresponding author: [scardenas@uco.es](mailto:scardenas@uco.es)

**1. Oral fluid collection**

30 min before the sample collection, the consumption of any food or drink was avoided, as well as chewing gum and teeth brushing. The cotton roll was introduced into the donor’s mouth until completely permeation (approximately 2-3 min). This sponge was transferred back into its container, where it was centrifuged 2 min at 3,000 rpm. The saliva samples were stored at 4ºC until analysis. Due to the SARS-CoV-2 global pandemic situation, the oral fluid samples were only donated by a healthy volunteer who was not medicated with TCAs.

**2. Mass spectrometry parameters**

Identification and quantitation were performed on an Agilent 6420 Triple Quadrupole MS with an electrospray source. The mass spectrometer settings were fixed to improve the multiple reaction monitoring (MRM) signals (Table S1). For TCAs, the flow rate and the temperature of the drying gas (N_2_, 99% purity) were 9 L/min and 300 ºC, respectively. The nebulizer pressure was 40 psi, and the capillary voltage was kept to 2000 V in positive mode. For acetaminophen, the flow rate and the temperature of the drying gas (N_2_, 99% purity) were 3 L/min and 300 ºC, respectively. The nebulizer pressure was 15 psi, and the capillary voltage was kept to 2000 V in positive mode. Agilent MassHunter Software (Version B-06.00) was used for qualitative and quantitative analyses.

**Table S1**. Multiple reaction monitoring transitions of the six analytes and the three internal standard compounds.

| Analyte | Precursor ion (m/z) | Product ion (m/z) | Q1 voltage | Collision energy (eV) |
| --- | --- | --- | --- | --- |
| Clomipramine | 315.2 | 86.1 (Q) | 114 | 18 |
|  |  | 58.2 |  | 50 |
| Trimipramine | 295.2 | 100.2 (Q) | 125 | 20 |
|  |  | 58.2 |  | 40 |
| Imipramine | 281.2 | 86.1 (Q) | 96 | 18 |
|  |  | 58.2 |  | 50 |
| Amitriptyline | 278.2 | 91.1 (Q) | 140 | 40 |
|  |  | 233.1 |  | 20 |
| Desipramine | 267.1 | 72.2 (Q) | 145 | 15 |
|  |  | 44.2 |  | 20 |
| Nortriptyline | 264.2 | 91.1 (Q) | 96 | 30 |
|  |  | 105.1 |  | 22 |
| Clomipramine-d3 | 318.2 | 89.2 (Q) | 132 | 18 |
|  |  | 61.2 |  | 46 |
| Desipramine-d3 | 270.2 | 75.2 (Q) | 132 | 18 |
|  |  | 193.0 |  | 42 |
| Nortriptyline-d3 | 267.2 | 91.1 (Q) | 96 | 30 |
|  |  | 105.1 |  | 22 |
| Acetaminophen | 151.9 | 110.1 (Q) | 115 | 30 |
|  |  | 65.2 |  | 20 |

Q: quantitation transition

**3. Effect of the wood matrix components on the analytical signal.**

Blank water samples were incubated with the WTs and the resulting solutions were spiked with the analytes at 20 µg/L. These solutions were finally analyzed by DI-MS/MS and the results are shown in Figure S1. For comparative purposes, the signal provided by a standard prepared in Milli-Q water at the same concentration level is included.


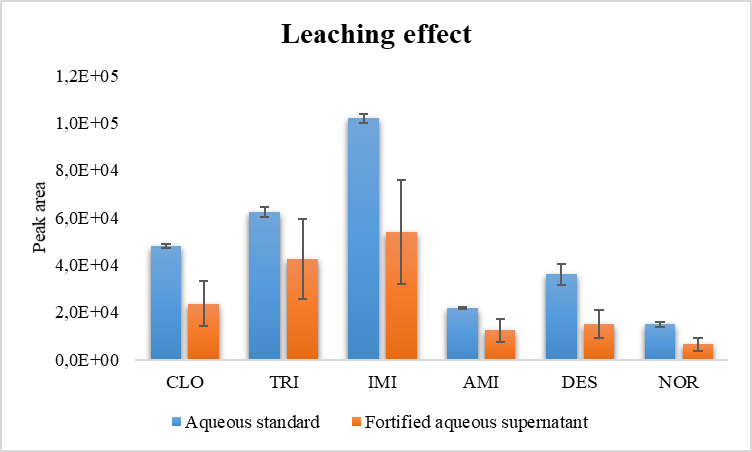


**Figure S1**. Effect of the wood matrix components on the analytical signal.

**4. Effect of the extraction time on the analytical signal.**


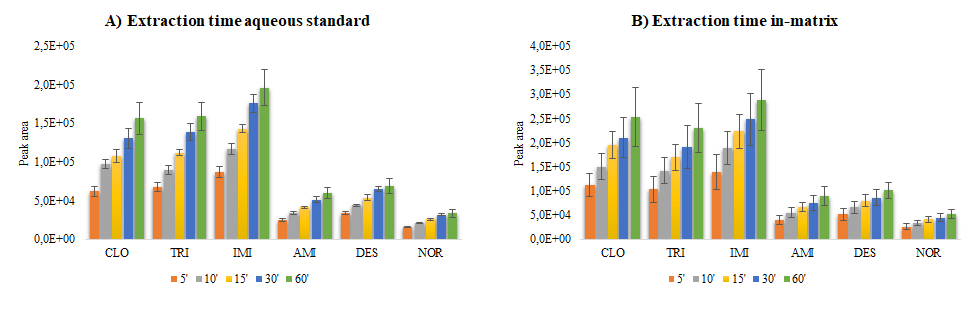


**Figure S2**. Effect of the extraction time on the analytical signal. Panels A shows how the absolute signal varies with the extraction time using aqueous standards. Panels B shows how the absolute signal varies with the extraction time using using blank oral fluid samples spiked with the target analytes.

**5 Potential of pre-cleaned WTs for in-vivo sampling**

The pre-cleaning of the WTs with boiling water maintains their biocompatibility derived from the lignocellulosic nature. This characteristic opens the door to the use of pb-WTs as sampling devices in oral fluid analysis. As no positive samples for antidepressants were obtained, acetaminophen was used as a model compound for in-vivo sampling. Acetaminophen is a worldwide drug used as a pain reliever and antipyretic. In this study, the dosage did not exceed the medically recommended values. Acetaminophen was taken as a headache reliever, and therefore the drug was not misused.

A volunteer took a pill of acetaminophen (1g) orally with water, and the mouth was then rinsed with 100 mL of water to remove potential residues of the drug. After that, a pre-cleaned WT was introduced in the mouth and sucked for 5 min. The pb-WT was collected, washed with Milli-Q water, and eluted in 200 µL of methanol. The eluate was finally analyzed by DI-MS/MS using the characteristic transition for acetaminophen (Table S1). This in-vivo sampling was repeated at different times. Figure S3A shows how the acetaminophen signal varies with time, obtaining the maximum signal at 45 min. This assay demonstrates the potential of the pre-cleaned WTs for in-vivo analysis. In fact, if not cleaned WTs are used the signal obtained for the analyte is completely suppressed (Figure S3B).


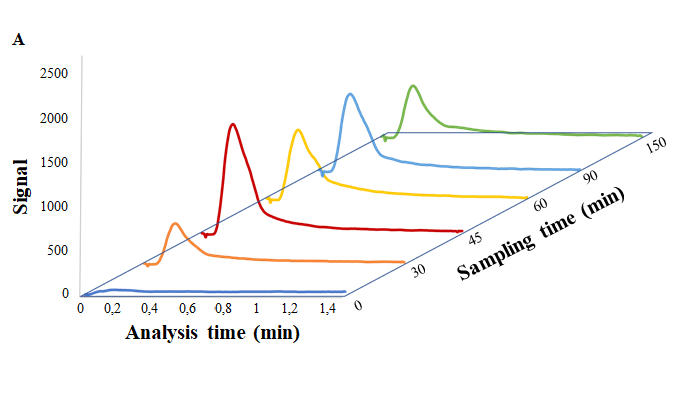

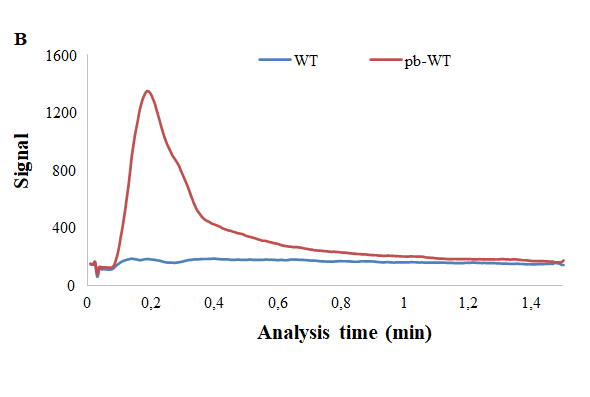


**Figure S3**. In-vivo sampling using acetaminophen. A) Effect of the sampling time using pb-WTs. B) Comparison of the signal using a WT and a pb-WT at 45 min of sampling time.
